# Antioxidant and Cytotoxic Constituents from *Wisteria sinensis*

**DOI:** 10.3390/molecules16054020

**Published:** 2011-05-17

**Authors:** Mona A. Mohamed, Manal M. Hamed, Allia M. Abdou, Wafaa S. Ahmed, Amal M. Saad

**Affiliations:** Department of Medicinal Chemistry, Theodor Bilharz Research Institute, P.O.B. 12411, Giza, Egypt; Email: manalayman@hotmail.com (M.M.H.); Allia.Mahmoud@yahoo.com (A.M.A.); eb2003eg@hotmail.com (W.S.A.); Amal_badawy@hotmail.com (A.M.S.)

**Keywords:** *Wisteria sinensis*, fabeaceae, acylated flavone, cytotoxic, Hep-G2

## Abstract

Chromatographic separation of an aqueous MeOH extract of *Wisteria sinensis* leaves has yielded six known flavonoids, two triterpene aglycones and the new acylated flavone glycoside chrysoeriol-7-*O*-[2''-*O*-(5'''-*O*-caffeoyl)-*β-*D-apiofuranosyl]-*β*-D-glucopyranoside (**1**). All metabolites were isolated for the first time from the genus *Wisteria*. Their structures were established on the basis of their chromatographic properties, chemical and physicochemical methods including acid hydrolysis analysis, spectroscopic (UV, ^1^*H*- and ^13^*C*-NMR) data and confirmed by ESI-MS analysis, as well as two-dimensional NMR (^1^*H*-^1^HCOSY, HMQC and HMBC). Biological studies of the defatted aqueous 80% methanol leaf extract and the major isolates **1**, **6** and **7** were undertaken and they are reported herein for the first time to have significant cytotoxic activity against the Hep-G2 tumor cell line in addition to antioxidant activity.

## 1. Introduction

*Wisteria* is a genus of flowering plants belonging to the pea family (Fabaceae), that includes ten species of woody climbing vines native to the eastern United States and the East Asian countries of China, Korea, and Japan. It is also an extremely popular ornamental plant in China and Japan. *Wisteria sinensis* (Sims.) DC. (Chinese wisteria), is a climbing plant that can reach a height of 20 m.

It is considered an invasive species in certain areas [1]. Its pendent racemes, with their many high blue-violet scented flowers, make a striking picture. The flowers of *W*. *sinensis* are also cured in sugar then mixed with flour and made into a famous local delicacy called "Teng Lo”. The leaves and flowers are also used as a tea substitute [2]. In addition the fiber from its stems can be used to make paper [3]. *Wisteria* species are used as a food source by the larvae of some *Lepidoptera* species of moth including the brown-tail [4]. Interestingly, many oriental medicinists use *Wisteria* gall extracts for treating gastric cancer [5] and cancer of breast and stomach, or rheumatoid arthritis patients [6,7,8,9,10,11,12,13]. Several *Wisteria* species have been also reported to have antioxidant [14] and antibacterial activities [15]. A survey of the literature showed that in previous studies phenylpropanoids and *β*-chromenes have been isolated from the oil of *W*. *sinensis* flowers [16] and several *Wisteria* species have been found to contain triterpene saponins, [6,7,8], isoflavones [10,11,12,13], and lectins [17,18,19]. To the best of our knowledge, there are no previous phytochemical investigations of this plant, thus we report herein our phytochemical studies on a methanol extract of *W. sinensis* leaves and evaluate the anticancer activity against Hep-G2 tumor cell lines and antioxidant activities of the investigated extract and its major isolates **1**, **6** and **7**.

## 2. Results and Discussion

Based on chemical and physicochemical analyses, compounds **2-9 **were identified as orientin (**2**), isoorientin (**3**), vitexen (**4**), isovitixen (**5**), apigenin (**6**), luteolin (**7**), [20,21,22,23], oleanolic acid (**8**) and hedragenin (**9**), respectively [24].

Compound **1**, was determined to be a chrysoeriol caffeoyl diglycoside on the basis of its chromatographic properties, UV spectra and acid hydrolysis results. Its UV spectrum (in methanol) exhibited a characteristic band I at ≈ 330 nm, along with a band II masked by an extra strong maximum at 262, 272 nm, which was indicative to the presence of a cinnamoyl moiety [23]. Upon complete acid hydrolysis of **1**, glucose and apiose were detected in the aqueous phase, while caffeic acid was identified along with chrysoeriol in the organic phase. The negative ESI-MS of **1 **exhibited a molecular ion peak at *m/z* 755.30 [M-H]^-^ corresponding to a molecular weight of 756, together with five diagnostic fragment ion peaks at *m/z* 593.46 [M-H-deoxycaffeoyl]^–^, 575.3 [M- H *-*deoxycaffeoyl-H_2_O]^–^, *m*/*z* 461.0 [M-H- deoxycaffeoyl apioside]^–^, *m*/*z* 299.4 [aglycone-H]^– ^corresponding to the loss of a glucoside from the last fragment and *m*/*z* 179.1 [caffeiate]^–^. Its ^1^H-NMR spectrum (Table 1) showed signals at 7.50 (1H, *brs*), 7.34 (1H, *dd*, *J* = 8.0, 1.8 Hz) and 6.68 (1H, *d*, *J* = 8.0 Hz) assigned to H-2', H-6' and H-5' respectively, in 1', 4'-disubstituted B-ring, two doublets at 6.67 (1H, *d*, *J* = 2.0 Hz) and 6.35 (1H, *d*, *J* = 2.0 Hz), attributed to H-8 and H-6, in 5,7- disubstituted A-ring, and one singlet at 6.55 (1H, *s*) attributed to H-3 together with a methoxy at 3.73 (3H, *s*). All the previous described signals were confirmative for a chrysoeriol moiety due to the deshielded location of H-2' (~+0.2 ppm). An *E*-caffeoyl moiety was deduced in the structure of **1** through an AX spin coupling system of two *E*-olefinic doublets at 7.29 H-7"" and 6.18 H-8"" (each, *d*, *J* = 16 Hz), along with ABX spin coupling system of its phenyl protons at 6.87 (*d*, *J* = 1.8 Hz), 6.84 *(dd*, *J* = 8.0, 1.8 Hz) and 6.67 (*d*, *J* = 8.0 Hz) of H-2"", H-6"" and H-5"", respectively. A doublet signal at 5.19 (1H, *d*, *J* = 7.5 Hz, H-1'') and a singlet at 5.37 (1H, *s*) in the ^1^H-NMR spectrum revealed the presence of two anomeric hydrogens from two sugar moieties. The ^13^C-NMR experiment showed 36 signals, of which 16 were attributed to the aglycone moiety, nine to the *E*-caffeoyl moiety, six to the *β*-D-glucopyranosyl moiety, and five possibly belonging to an apiofuranosyl moiety. 

**Table 1:** ^1^H-, ^13^C-NMR and HMBC spectral data of **1** (500/125 MHz, DMSO-*d_6_*).

NO	δH	δC	HMBC correlation
**2**		164.3	
**3**	6.55 *s*	103.7	
**4**		181.7	
**5**		161.2	
**6**	6.35 *d* (2.0)	99.5	H-8, 10
**7**		162.3	
**8**	6.67 *d* (2.0)	94.3	H-6, 10
**9**		156.7	
**10**		105.4	
**1'**		121.6	
**2'**	7.50 (*brs*)	113.7	H-6', 4'
**3'**		147.6	
**4'**		149.3	
**5'**	6.68 *d* (8.0)	115.0	H-3', 1'
**6'**	7.34 *dd* (8.0, 1.8)	115.4	H-2', 4'
**OCH_3_**	3.73 *s*	55.5	H-3'
**1''**	5.19 *d* (7.5)	99.1	H-7, 3''
**2''**	3.78 *dd* (10.5, 7.5 )	75.8	H-1''', 4''
**3''**	3.41 *brd*	76.6	H-5'', 1''
**4''**	3.20 *t* (10.5)	69.8	H-2'', 6''
**5''**	3.46 *m*	77.5	H-3''
**6''a**	3.52 *m*	60.5	
**6''b**	3.70 *brd* (12.0)	60.5	
**1'''**	5.37 *s*	108.2	H-3''', 2''
**2'''**	3.58*	76.7	H-4'''
**3'''**	3.65*	77.0	H-1'''
**4'''a**	3.75 *d* (9.5)	73.8	
**4'''b**	4.04 *d* (9.5)	73.8	
**5'''**	4.07 *brs*	66.6	H-2'''
**1''''**		125.4	
**2''''**	6.87 *d* (1.80)	115.4	H-6'''', 4'''', 7''''
**3''''**		145.0	
**4''''**		149.3	
**5''''**	6.67 *d* (8.0)	115.9	H-3'''', 1''''
**6''''**	6.84 *d* (8.0, 1.8)	122.8	H-2'''', 4'''', 7''''
**7''''**	7.29 *d* (16.0)	144.9	H-5'''', 2'''', 9''''
**8''''**	6.18 *d* (16.0)	113.4	H-1''''
**9''''**		167.5	

* Unresolved proton resonances, δ in ppm and *J* values (Hz), were given in parentheses. All carbon and proton resonances were assigned on the basis of 2D (^1^H-^1^H COSY, HMQC and HMBC).

The apiose moiety was characterized through its one bond correlation in the HMQC spectrum with its own proton signals compared to the literature data [25,26,27]. The glucosyl and apiosyl sugar moieties were deduced to adopt *β*-D-pyranosyl and *β*-D-furanosyl stereostructures, respectively, on the basis of *J*-values of their anomeric protons and δ-values of their ^1^H and ^13^C-resonances (Table 1). 

**Figure 1:**
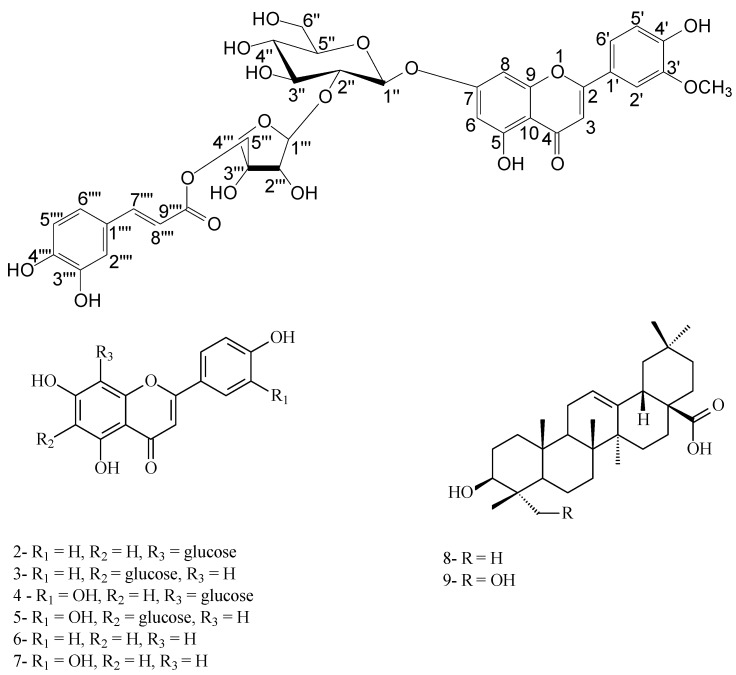
Chemical structures of isolated compounds from the leaves of *Wisteria sinensis*.

Additional evidence for the chrysoeriol moiety was obtained from the deshielded shift of C-3' (~ +3 ppm) (Table 1) and the observation of the three bond correlation peak between the methoxy proton signal at δ 3.73 and the C-3' signal at δ 147.6 in the HMBC spectrum. The glycosidation at 7-OH was concluded from the downfield shift of both C-6 and C-8 (≈ + ∆ 0.2 ppm) and long-range correlation between the hydrogen signal at 5.19 (H-1'' glucose) and the carbon signal at 162.3 (C-7 aglycone) in the HMBC spectrum. Besides, the chemical shift of the C-2'' of the glucose moiety at 75.8 was clearly deshielded (≈ + ∆3 ppm) compared to the chemical shift of the analogous carbon resonance of a non-substituted moiety (72.9), led us to identify the glycoside moiety as 7-*O*-*β*–D-glucopyranosyl (1'"→2") apiofuranoside. This was further confirmed from HMBC experiments showing a long-range correlation between the hydrogen signal at 3.78 (H-2" glucose) and the carbon signal at 108.2 (C-1''' apiose). In addition, the esterification of the *E*-caffeoyl group to C-5"' was deduced from the characteristic downfield shift of C-5"' apiofuranosyl (66.6) relative to that of a non-acylated analogue (62.4), together with the long range correlation between this protons and the carbonyl carbon of the *E*-caffeoyl moiety (167.5) in HMBC spectrum. All other ^1^H- and ^13^C-NMR resonances were also confirmed by the ^1^H-^1^H-COSY, HMQC and HMBC spectra and by comparison with previously reported data for structurally related compounds [28,29,30,31,32]. Thus, **1 **was identified aschrysoeriol -7-*O*-[2''-*O*-(5'''-*O*-caffeoyl)-*β*-D-apiofuranosyl]-*β*- D-glucopyranoside (Figure 1). 

**Figure 2:**
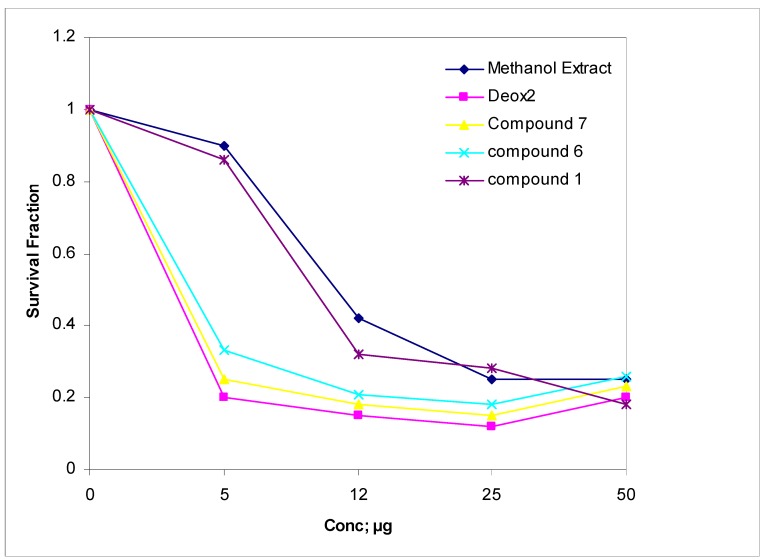
The cytotoxic activity of *W. sinensis* MeOH extract, **1**, **6** and **7** against HepG2 cell line. DOX2 = doxorubicin (positive control).

Regarding their IC_50_ values (Figure 2), all tested compounds and the methanol extract exhibited anticancer activity against HepG2 cells in the order **7**, **6**, **1** and methanol extract. The anti-cancer activity of *W. sinensis* leaf methanol extract may be attributed to the corresponding activities of the flavonoid extract constituents. Experimental animal studies indicate that certain flavonoids possess antitumor activity. The hydroxylation pattern of the B ring of the flavones, such as luteolin and quercetin, seems to critically influence their activities, especially the inhibition of protein kinase antiproliferation activity [33]. Luteolin (**7**) is an important member of the flavonoid family. It has been reported that this substance can inhibit the proliferation of a variety of tumor cells, including solid tumors, ascties cancer and human myeloid leukemia. Luteolin can also sensitize a number of apoptosis-inducing factors by unique mechanisms [34]. 

**Figure 3:**
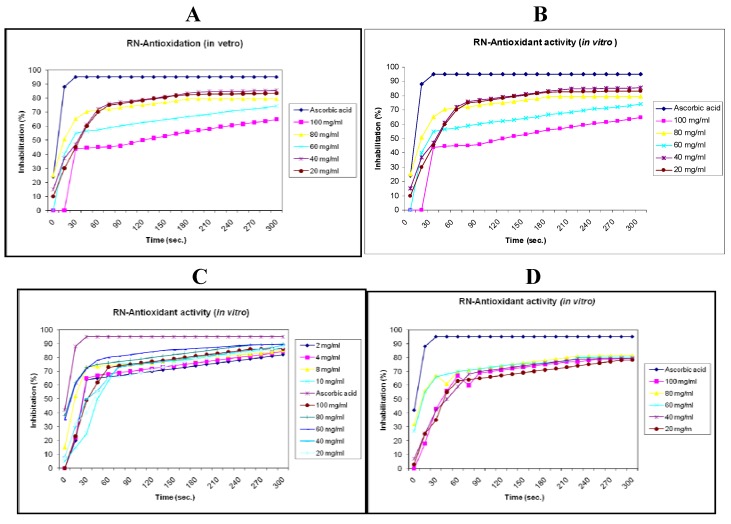
**A**. Antioxidant activity of methanol extract; **B**. Antioxidant activity of **1**; **C.** Antioxidant activity of **6**; **D.** Antioxidant activities of **7**.

The antioxidant properties of flavonoids are widely acknowledged [35,36,37]. The two classical structural antioxidant features of flavonoids are the presence of a B-ring catechol group and the presence of a C_2_-C_3_ double bond in conjugation with an oxo group at C4; the first serves to donate hydrogen/electron to stabilize a radical species and the second serves to bind transition metal ions such as iron and copper [37,38]. Because luteolin and some of its glycosides fulfill both structural requirements, it is not surprising that many luteolin-containing plants possess antioxidant properties [39]. The antioxidant activity of luteolin and its glycosides has been associated with their capacity to scavenge reactive oxygen and nitrogen species [40], to chelate transition metals that may induce oxidative damage through the fenton reaction [38], to inhibit proxidant enzymes [39,41,42] and to induce antioxidant enzymes [43,44,45]. The antioxidant activity of luteolin has not only been observed *in vitro* but also *in vivo* [46]. In line with this observation, all tested compounds and methanol extract exhibited strong antioxidant activity (Table 2 and Figure 3).

**Table 2:** Maximum reactive reaction rate after 5 min. for compounds **1**, **6** and **7** at different concentrations.

Investigated materials	Maximum reactive reacting rate after 5 min concentration mL^−1^								
	2	4	8	10	20	40	60	80	100
Methanol extract	-	-	-	-	82.9	861	74.5	79.4	65.1
1	-	-	-	-	86.1	88.6	89.1	88.7	87.1
6	-	-	-	-	77.9	79.7	81.1	82.5	87.3
7	80.5	83.1	84.2	85.3	79.7	81.5	82. 9	82. 9	81.5

## 3. Experimental

### 3.1. General

NMR spectra for known compounds were recorded at 300 (^1^H) and 75 MHz (^13^C) on a Varian Mercury 300, while those of the new compound **1** were recorded at 500 (^1^H) and 125 MHz (^13^C) on a JEOL GX-500 NMR spectrometer and δ values are reported in ppm relative to TMS in the corresponding solvent. ESI-MS analyses were measured on Finnigan LCQ deca LC/MS and double focusing sector field MAT 90 MS spectrometers (Finnigan, Bremen, Germany). UV analyses of pure samples were recorded, separately, in MeOH solutions on a Shimadzu UV 240 spectrophotometer. 

### 3.2. Plant material

Leaves of *Wisteria sinensis* were collected in Spring from plants growing in the El-Orman Botanical Garden, Giza, Egypt. The plant was authenticated by Prof. Wafaa M. Amer, Department of Botany, Faculty of Science, Cairo University, Giza, Egypt. A voucher specimen (Reg. No.: P–I) was deposited in the herbarium, Medicinal Chemistry Department, Theodor Bilharz Research Institute, Giza, Egypt.

### 3.3. Extraction and isolation

Air-dried ground powders leaves of *W. sinensis* (750 g) were extracted with hot 85% aqueous methanol (4 × 3 L) under reflux (70 °C). The extract was then dried under reduced pressure and redissolved in methanol. The methanol-soluble portion (75 g) was suspended in water and preliminarily fractionated on a Polyamide S column (Ø 7 × 110 cm, 350 g, Fluka, St. Louis, MO, USA), using a step gradient from water 100% to methanol 100% for elution to yield 56 fractions of 1 L each, which were concentrated under reduced pressure. On the basis of comparative thin layer chromatography (TLC) and paper chromatography (PC) using solvent systems S_1_, S_2_ and S_3_ [S1, *n*-butanol–acetic acid– water (4:1:5, top layer), S_2_, 15% aqueous acetic acid and S_3_, CHCl_3 _- MeOH (8:2)] with the use of UV light, 10% H_2_SO_4_, FeCl_3_ and Naturstoff spray reagents, the 56 individual fractions were grouped into four major fractions (I–IV). The dry material of fraction I was redissolved in methanol and filtered to remove some free sugars and salts. A part of the methanol soluble portion of fraction I (320 mg) was subjected to repeated column chromatography (CC) on Sephadex LH-20 (Pharmacia, Uppsala, Sweden) and eluted with methanol to give pure **1** (65 mg). Fraction II (eluted with 30%–60% methanol, 9 g) was subjected to CC on Sephadex LH-20 twice, followed by individual precipitation of both main subfractions from ethanol by excess ethyl acetate to give pure samples of **2** (19 mg) and **3** (20 mg). Fraction III (eluted with 60%–80% methanol, 12 g) was fractionated on cellulose C (Merck) with 60% MeOH as an eluent, followed by a Sephadex LH-20 column using BIW (*n*-BuOH/2-propanoI/H_2_O, 4:1:5 v/v/v, organic layer) to afford pure **4** (27 mg) and **5** (22 mg). Fraction IV, (80%–100% methanol, 15 g) gave two dark purple spots on PC under long UV, which change to dark green color with the use of FeCl_3 _and two pink color spots with sulphuric acid spray reagent on TLC when heated at 120 °C for three min. Fraction IV was dried under vacuum and was subjected to separation over a silica gel 60 column (Sigma, 28–200 mesh, Ø 3.0 × 50 cm, 100 g) using a gradient of CHCl_3_-MeOH (8:2, 7:3, 1:1, 4:6 and 0:1) to give 20 individual fractions of 75 mL each. On the basis of comparative TLC and PC using the previously described solvent systems, the individual 20 fractions were pooled in two collected fractions (A and B) according to the differences in composition. Fraction A eluted with (20%-30% MeOH in CHCl_3_) was subjected to (CC) on Sephadex LH-20 to give pure samples of **6 **(55 mg) and **7 **(63 mg). Pure **8 **(24 mg) and **9 **(32 mg) were isolated from fraction B (30%–100% MeOH in CHCl_3_) using a Sephadex column and EtOH for elution. All separation processes were followed up by comp-PC and TLC using S_1_, S_2_ and S_3_. Silica gel powder (F_254_, Merck; Darmstadt, Germany) were used for TLC. For paper chromatography Whatman No. 1 sheets (Whatman Ltd., England) were used. All aglycones and sugars obtained by acid hydrolysis were identified by comp-TLC and PC with authentic samples, using the previously described solvent systems, in addition to EtOAc-C_5_H_5_N-H_2_O (12:5:4), and specific spray reagents (e.g., vanillin HCl and aniline hydrogen phthalate).

### 3.4. Chrysoeriol -7-O-[2''-O-(5'''-O-caffeoyl)-β-D-apiofuranosyl]-β- D-glucopyranoside (***1***)

Yellow amorphous powder**, **Rf-values: 0.73 (S1), 0.77 (S2). Negative ESI-MS: *m/z* 755.3[M-H]^−^, 593.46 [M-H- deoxycaffeoyl]^−^, 575.30 [M-H- deoxycaffeoyl-H_2_O]^−^, 461.0 [M-H-deoxycaffeoyl apioside]^−^, 299.4 [aglycone-H]^– ^, 179.1 [caffeiate]^−^. ^1^H- and ^13^C-NMR: see Table 1. 

### 3.5. Measurement of potential cytotoxicity by SRB assay

Potential cytotoxicity of the methanol extract of *W. sinensis* leaves and the isolated compounds **1**, **6**, **7** and doxorubicin as positive control and transformed cells as a negative control were tested at the National Cancer Institute, Egypt, using the method of Skehan *et al.* [47]. Cells were plated in a 96-well plate (104 cells/well) for 24 h before treatment to allow the attachment of cells to the wall ofthe plate. Different concentrations of the materials under investigation (0, 1, 2.5, 5 and 10 μg/mL) were added to the cell monolayer. Triplicate wells were prepared for each individual dose and they were incubated for 48 h at 37 °C in 5% CO_2_. After 48 h cells were fixed, washed and stained with sulforhodamine B stain. Excess stain was washed with acetic acid and attached stain was recovered with Tris-EDTA buffer and the color intensity was measured in an ELISA reader. The average of the transformed cells was recorded. The survival curve of the tumor cell line was plotted for each tested fraction.

### 3.6. Antioxidant activity

This activity was investigated *in vitro* for the investigated methanol extract and compounds **1**, **6 **and **7** using 1,1-diphenyl-2-picrylhydrazyl (DPPH) free radicals, according to the method of Peiwu *et al.* [48]. In a disposable cuvette a methanolic solution of DPPH (2.95 mL) was added to samples of methanol extract and investigated compounds (50 µL in MeOH) at different concentrations (2–100 mg mL^−1^) for luteolin and (20–100 mg mL^−1^) for methanol extract and compounds **1 **and **6**. The absorbance was measured at 517 nm at regular intervals of 15 s for 5 min. Ascorbic acid was used as standard (0.1 M) as described by Govindarajan *et al.* [49].

## 4. Conclusions

There is a real need to identify new compounds with potential anticancer activity, so experiments exploring the anticancer potential of compounds from plants used in traditional medicines for the treatment of many cancer diseases are required for the isolation and development of new anticancer compounds. The compounds discussed in the present study have only been tested in a limited screen against Hep-G2 tumor cell line; therefore, further anticancer experiments on these compounds against other cell lines are required to explore their anticancer activities in greater detail. 
